# Case report: the first case of unilateral retinal pigment epithelium dysgenesis in China

**DOI:** 10.1186/s12886-020-01609-4

**Published:** 2020-08-20

**Authors:** Yuhua Ding, Bangtao Yao, Keren Xie, Hui Ye, Yan Yu

**Affiliations:** 1 Department of Ophthalmology, Jiangsu province hospital, The First Affiliated Hospital of Nanjing Medical University, Nanjing, Jiangsu Province China; 2 Department of Ophthalmology, Lishui District People’s Hospital, Lishui branch of Southeast University Affiliated Zhongda Hospital, Nanjing, Jiangsu Province China

**Keywords:** RPE, Dysgenesis, OCTA, En-face OCT, Multimodal imaging

## Abstract

**Background:**

Unilateral retinal pigment epithelium dysgenesis (URPED) is a rare condition and is characterized by a unilateral and solitary lesion in the peripapillary region. The lesion presents with central atrophy, peripheral fibrosis, and hyperplastic changes in the retinal pigment epithelium (RPE). Herein, we report the first case of URPED in a Chinese individual using multimodal imaging techniques such as en-face optical coherence tomography (OCT) and optical coherence tomography angiography (OCTA).

**Case presentation:**

A 10-year-old girl presented with 20/40 vision in her left eye. Presented as a solitary, unilateral, large and yellowish-white lesion, with fringe-like margins was observed in the posterior pole and lower middle periphery of the left eye continuous with the optic nerve, indicated URPED. Infrared fundal (IR) images showed that the fringe-like contour of the lesion was visible, with diffuse hyperreflective signals specifically in the fovea, while with peripheral dark spots, having a typical leopard-spot like appearance. Fundus autofluorescence (FAF) revealed a markedly scalloped lesion containing a hypoautofluorescence area mixed with an isoautofluorescence area. Spectral-domain optical coherence tomography (SD-OCT) revealed the outer segments of photoreceptors presented with an inhomogeneous signal in the fovea, with a weak local signal. The ellipsoid and interdigitation zones were thinner than normal, while the RPE/Bruch’s complex was not flat, with locally visible protrusions. En-face OCT image at the level of the RPE zone showed a mottled hyperreflective signal with peripheral hyporeflective spots, fringe-like margin lesions. OCTA of the avascular area of the fovea in the superficial, deep, and outer retinal layers appeared to be oval in shape, the choroid capillary layer revealed an increase in the density of the choroidal vasculature in the fovea.

**Conclusions:**

This is the first report on URPED in China. Both en-face OCT and OCTA were essential in observing and studying the disease. Further investigation is required to better define the en-face OCT and OCTA features of URPED and clarify the disease characteristics and prognosis.

## Background

Unilateral retinal pigment epithelium dysgenesis (URPED) is a rare entity and is characterized by a unilateral and solitary lesion in the peripapillary region. The lesion presents with central atrophy and peripheral fibrosis, along with hyperplastic changes in the retinal pigment epithelium (RPE) [1]. It was first described by Cohen et al. [2], and has been reported in about 22 case studies [1–10]. This is possibly the first study to present a case of URPED in China and to use optical coherence tomography angiography (OCTA) along with en-face mode of optical coherence tomography (OCT) to observe the same.

## Case presentation

**Fig. 1:**
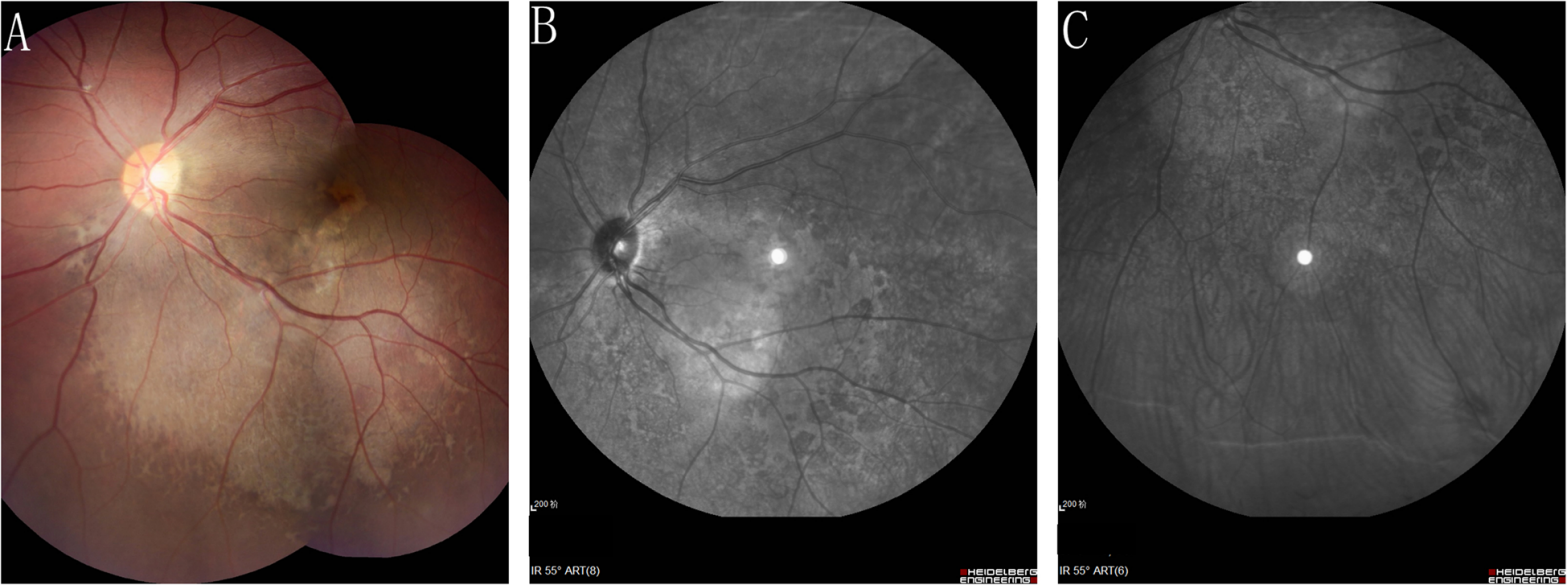
In the left eye: a solitary, unilateral, large, scalloped and yellowish-white lesion, with fringe-like margins was observed in the posterior pole and lower middle periphery of the left eye continuous with the optic nerve. Furthermore, we noted the presence of fibrosis and hyperplastic changes in the RPE at the periphery and thinning at the center of the lesion (**a**). IR images showed that the fringe-like contour of the lesion was visible, with diffuse hyperreflective signals specifically in the fovea, while with peripheral dark spots, having a typical leopard-spot like appearance (**b**, **c**)

**Fig. 2:**
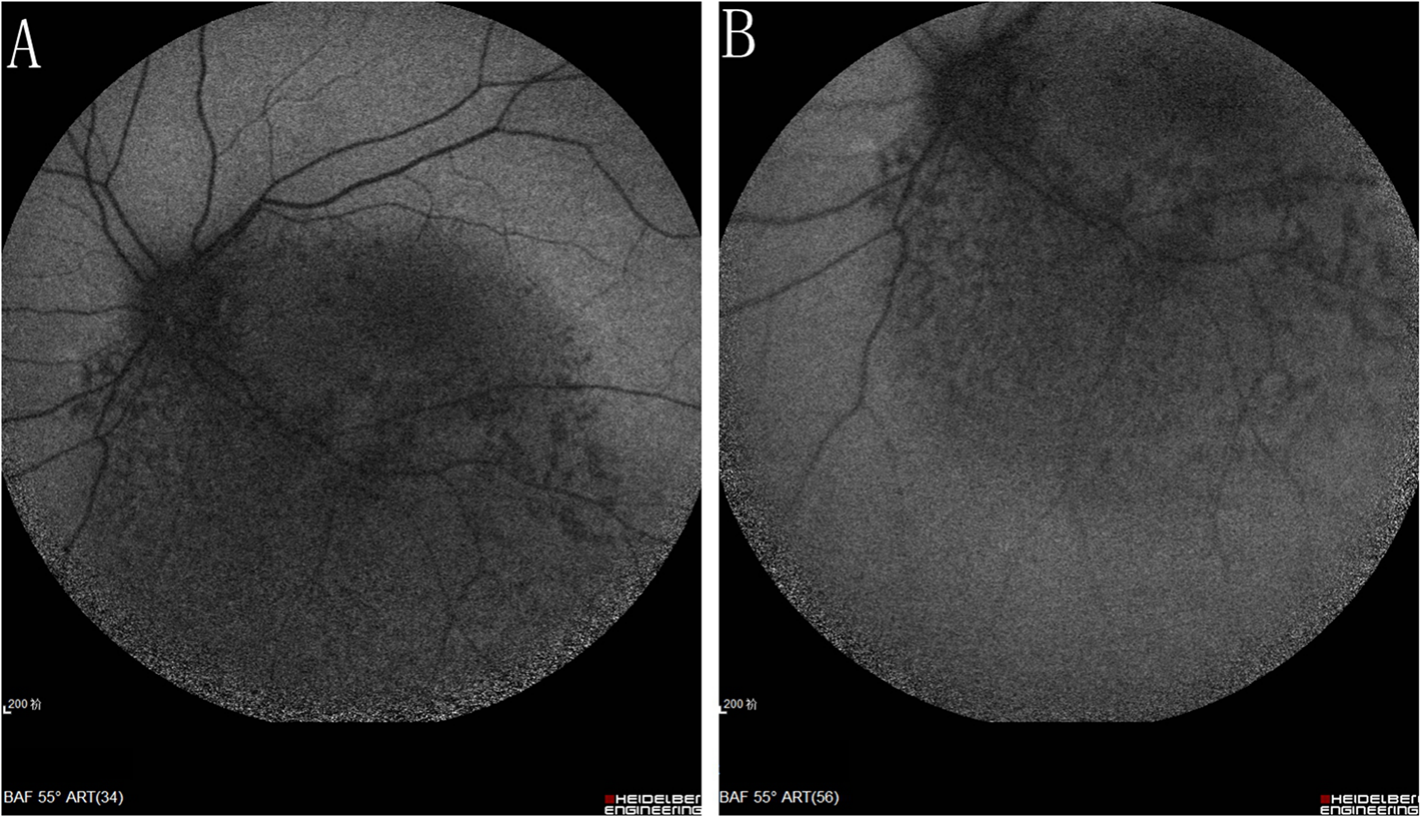
FAF revealed a markedly scalloped lesion containing a hypoautofluorescence area mixed with an isoautofluorescence area (**a**, **b**)

**Fig. 3:**
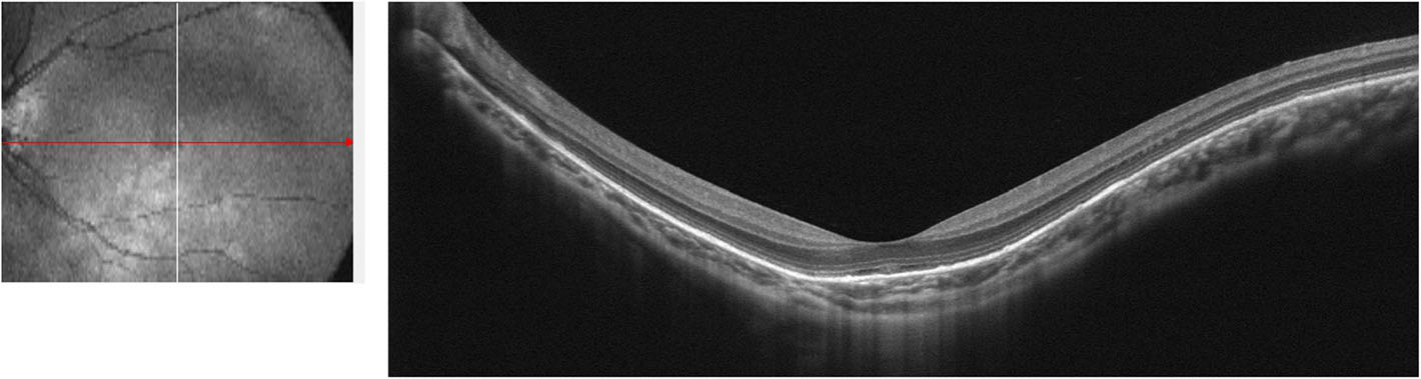
SD-OCT revealed certain unique characteristics of the same. The outer segments of photoreceptors presented with an inhomogeneous signal in the fovea, with a poor local signal. The ellipsoid and interdigitation zones were thinner than normal, while the RPE/Bruch’s complex was not flat, with locally visible protrusions (Fig. 3)

**Fig. 4:**
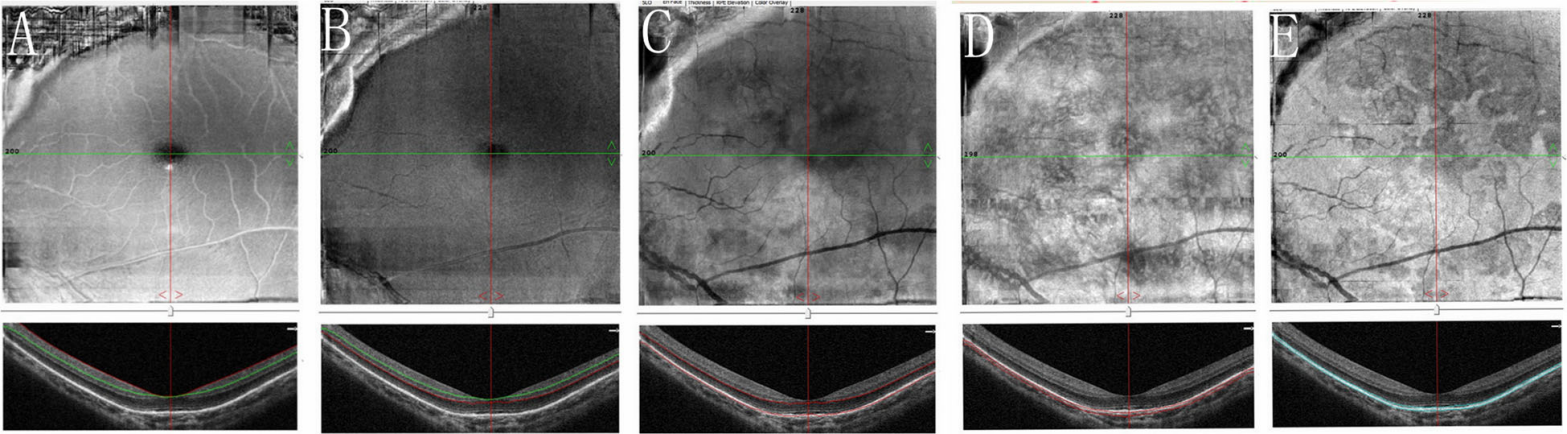
En-face OCT image at the level of the superficial (**a**) and deep retinal layers (**b**) appeared normal, the outer retinal layer (**c**), and the choroid capillary layer (**d**) revealed a weak mottled hyperreflective signal; the RPE zone showed a mottled hyperreflective signal with peripheral hyporeflective spots, fringe-like margin lesions (**e**)

**Fig. 5:**
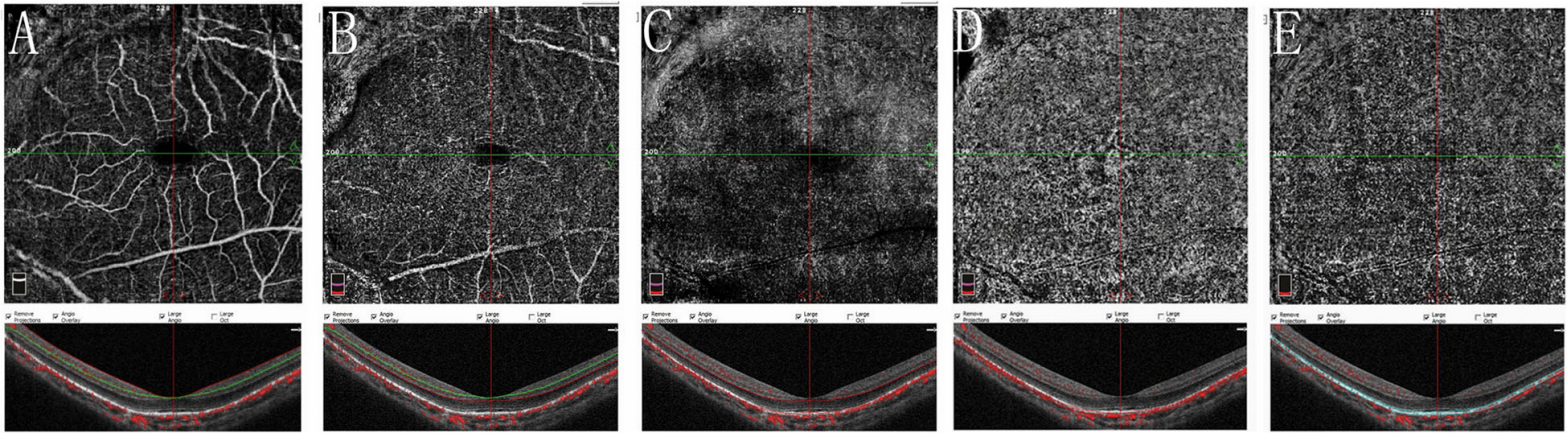
OCTA of the avascular area of the fovea in the superficial (**a**), deep (**b**), and outer (**c**) retinal layers appeared to be oval in shape, the choroid capillary layer revealed an increase in the density of the choroidal vasculature in the fovea (**d**); OCTA image at the level of the RPE zone appeared normal (**e**)

**Fig. 6:**
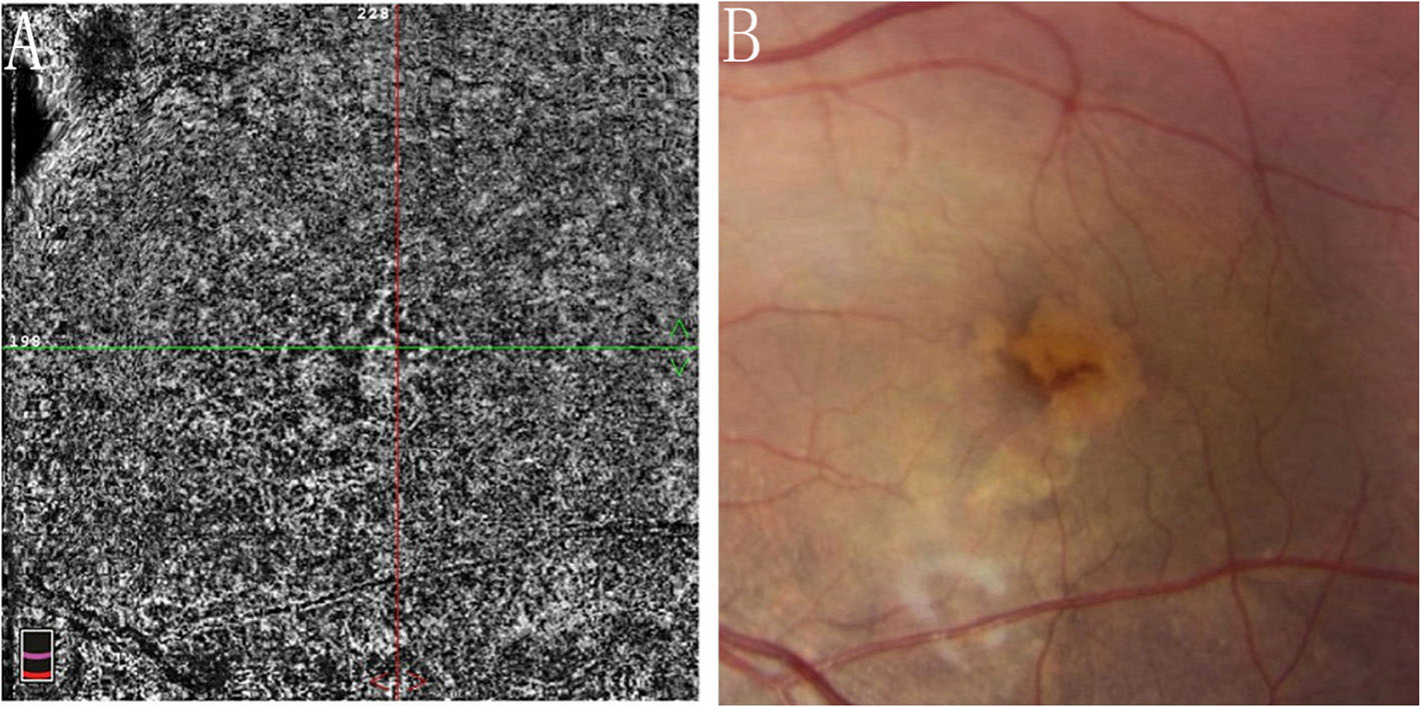
The density of the packed honeycomb structure in the central macula increased in choroid capillary layer (**a**) was consistent with the central atrophy yellow-whitish lesion in the fundus photograph (**b**), which revealed a thinner RPE for increased density of the choriocapillaris

A 10-year-old girl presented with a painless reduction in visual acuity in her left eye, incidental finding on the school routine examination. The fundus revealed a unilateral solitary lesion of the RPE with fibrosis and hyperplastic changes at its periphery and thinning in the center. No family or trauma history was noted. The medical and ophthalmic history of the patient were negative. General pediatric physical examination results were normal for all systems. The initial best-corrected visual acuity (BCVA) was 20/20 in the right eye and 20/25 in the left eye. The diopter of both eyes was 0 DS OD and, − 2.0 DS OS. The anterior segment and intraocular pressure were normal. Funduscopic examination of the right eye was unremarkable. A solitary, unilateral, large, scalloped and yellowish-white lesion, with fringe-like margins was observed in the posterior pole and lower middle periphery of the left eye continuous with the optic nerve. Furthermore, we noted the presence of fibrosis and hyperplastic changes in the RPE at the periphery and thinning at the center of the lesion (Fig. 1a). Infrared fundal (IR) images showed that the fringe-like contour of the lesion was visible, with diffuse hyperreflective signals specifically in the fovea, while with peripheral dark spots, having a typical leopard-spot like appearance (Fig. 1b, c). Fundus autofluorescence (FAF) revealed a markedly scalloped lesion containing a hypoautofluorescence area mixed with an isoautofluorescence area (Fig. 2a, b). Spectral-domain optical coherence tomography (SD-OCT) revealed certain unique characteristics of the same (Fig. 3). The outer segments of photoreceptors presented with an inhomogeneous signal in the fovea, with a weak local signal. The ellipsoid and interdigitation zones were thinner than normal, while the RPE/Bruch’s complex was not flat, with locally visible protrusions. En-face OCT image at the level of the superficial and deep retinal layers appeared normal (Fig. 4a, b), the outer retinal layer (Fig. 4c), and the choroid capillary layer (Fig. 4d) revealed a weak mottled hyperreflective signal; the RPE zone showed a mottled hyperreflective signal with peripheral hyporeflective spots, fringe-like margin lesions (Fig. 4e). OCTA of the avascular area of the fovea in the superficial, deep, and outer retinal layers appeared to be oval in shape (Fig. 5a, b, c), the choroid capillary layer revealed an increase in the density of the choroidal vasculature in the fovea (Fig. 5d), the density of the packed honeycomb structure in the central macula increased in OCTA was consistent with the central atrophy yellow-whitish lesion in the fundus photograph (Fig. 6); OCTA image at the level of the RPE zone appeared normal (Fig. 5e). Based on the clinical examination and characteristic multimodal imaging findings, the patient was diagnosed to have URPED.

The treatment for this patient was closely observed. At the last follow-up examination, after 18 months, there were no changes in the fundus, and her BCVA remained 20/20 OD and 20/25 OS.

## Discussion and conclusions

URPED is a rare, unilateral, benign, well-defined, and congenital RPE dysgenesis that was first described by Cohen et al. in 4 patients in 2002 [2]. They noted that the lesion appeared as a leopard-spot lesion contiguous with the optic nerve. Typically, the lesion located in the peripapillary region and is accompanied by central atrophy, peripheral fibrosis, and hyperplastic changes in the RPE. In 2009, Cohen et al. [1] further described this rare condition in a set of 9 affected patients, describing the FAF and OCT features. The hypoautofluorescent lesion with a hyperautofluorescent border was noted to be inverted when compared with the fundus fluorescein angiography (FFA) outcomes. This distinct inverted pattern on FAF and FFA was due to the pathognomonic clinical features of peripheral scalloped reticular margins and dark central RPE hyperplasia and atrophy.

The etiology of this condition remains unclear. Renz et al. [10] hypothesized that it might be an RPE dysgenesis or dystrophy, or may indicate previous inflammatory, infectious, or autoimmune responses to the RPE.

The lesion presented in the fundus photography is by far more pale than in previous cases published [4]. We considered that the pigmentation of the lesion may be less obvious in Asian patients or in young patients.

Autofluorescence signals are predominantly derived from lipofuscin within the RPE [11]. Here, FAF showed a marked lesion with an affected hypoautofluorescence area mixed with an isoautofluorescence area. The scalloped reticular margin of the lesion seen on FAF was consistent with the contours observed on the fundus photographs. Presence of hypoautofluorescence indicated PRE dysgenesis. Therefore, we speculated that the isoautofluorescence area could represent the normal functional RPE surrounding the impaired functional hypoautofluorescence RPE.

SD-OCT outcomes suggested that the outer segments of photoreceptors demonstrated an inhomogeneous signal in the foveal region, with a weak local signal. The ellipsoid zone and interdigitation zone were thinner than normal. The RPE/Bruch’s complex was not flat, and demonstrated locally visible protrusions that may have negatively influenced the patient’s BCVA. Cohen et al. [1] observed central atrophic changes in the RPE in each of the five cases, which was consistent with our observation.

In normal eyes, structural en face OCT image on the RPE layer is homogeneous and hyperreflective, which makes it easier to distinguish from the surrounding and better resolved tissues. The only hyporeflective elements at this level are the optic disc and the tomographic shadowing of the normal retinal vessels above [12]. In our case, the en-face OCT image at the level of the retinal pigment epithelial zone showed a large, vivid, and scalloped lesion, containing a mottled hyperreflective signal with peripheral hyporeflective spots and fringe-like margins. This information from en-face OCT also strongly supported the fact that the lesion originated from the RPE layer. The outer retinal layer and the choroid capillary layer revealed that the weak mottled hyperreflective signal was an artificial signal caused by the interference of the RPE signal and influence of manual stratification, while the superficial and deep layers appeared to be normal.

OCTA is a recently developed technique that facilitates non-invasive visualization of vascular structures. It was proven to document in vivo choriocapillaris and choroidal vasculature in a reproducible and non-invasive manner, which is an invaluable tool to examine the choroid in either physiological or pathological conditions [13]. Microvasculature patterns and densities can be observed using OCTA. Here, the density of the packed honeycomb structure in the central macula of choroid capillary layer increased in OCTA was consistent with the central atrophy yellow-whitish lesion in the fundus photograph, which revealed a thinner RPE for increased density of the choriocapillaris. The avascular area of the fovea appeared to become oval in shape in the superficial, deep, and outer layers, possibly due to the longer axis secondary to the refractive error of − 2.0 DS.

Despite the very distinctive fundus features of URPED, it is important to address other similar clinical differential diagnoses. First, peripapillary acute zonal occult outer retinopathy (AZOOR) may appear as a rounded or U-shaped whitish lesion around the optic disc [14]. In peripapillary AZOOR, FAF of the lesion presents as an atrophic hypoautofluorescent pattern, while its outer margin is intense hyperautofluorescence in a linear shape, which is significantly different from the reticular, fringe-like margin in URPED. Second, traumatic retinal pigment epitheliopathy and resolution of hemorrhagic detachment of the retina [15]. Third, combined hamartoma of the retina and RPE (CHRRPE) is a rare, benign, probably congenital, intraocular tumor that characteristically presents as a unilateral pigmented but obviously elevated mass, often with wrinkling and disorganization of the retina, tortuous overlying vessels, and vitreoretinal interface changes [16]. Furthermore, these clinical manifestations, medical history, and FFA differ greatly from URPED. Fourth, Choroidal osteoma is a rare benign ocular tumor characterized by an ossifying lesion within the choroid that is well defined, and tends to occur unilaterally [17]. The two diseases have completely different boundaries. URPED presents large, scalloped and yellowish-white lesion, with fringe-like margins, while choroidal osteoma presents well defined margin. Besides, in the examination of the fundus of osteoma, there was a prominent elevated mass, which is distinct with URPED.

However, this study has several limitations. We lacked the FFA information due to the child’s parents did not agree to perform the examination. The sample size of the study was relatively small. Additionally, the follow-up time needed to be longer since the disease reportedly probably progresses gradually [6].

In conclusion, this is the first report on URPED in China, both en-face OCT and OCTA are useful tools to observe the disease. The processes associated with the natural development of URPED remain unclear. Further studies and longer follow-up times would be required to better define en-face OCT and OCTA features of URPED and clarify the characteristics and development of this rare disease.

## Data Availability

The datasets used and/or analyzed during the current study are available from the corresponding author on reasonable request.

## Abbreviations

AZOOR: Acute zonal occult outer retinopathy
BCVA: Best-corrected visual acuity
CHRRPE: Ccombined hamartoma of the retina and RPE
FAF: Fundus autofluorescence
FFA: Fundus fluorescence angiography
IOP: Initial intraocular pressure
IR: Infrared fundal
OCTA: Optical coherence tomographic angiographic
RPE: Retinal pigment epithelium
SD-OCT: Spectral-domain optical coherence tomographic
URPED: Unilateral retinal pigment epithelium dysgenesis
